# Methyl *N*-phenyl­succinamate

**DOI:** 10.1107/S160053680904690X

**Published:** 2009-11-11

**Authors:** B. Thimme Gowda, Sabine Foro, B. S. Saraswathi, Hartmut Fuess

**Affiliations:** a Department of Chemistry, Mangalore University, Mangalagangotri 574 199, Mangalore, India; b Institute of Materials Science, Darmstadt University of Technology, Petersenstrasse 23, D-64287 Darmstadt, Germany

## Abstract

In the structure of the title compound, C_11_H_13_NO_3_, the conformations of the N—H and C=O bonds in the amide fragment are *trans* to each other. In the crystal, mol­ecules are linked into a 2_1_ helical chain that propagates along the *c* axis through N—H⋯O inter­actions.

## Related literature

For related structures, see: Gowda *et al.* (2007 ▶, 2009**a* ▶,b* ▶); Jones *et al.* (1990 ▶).
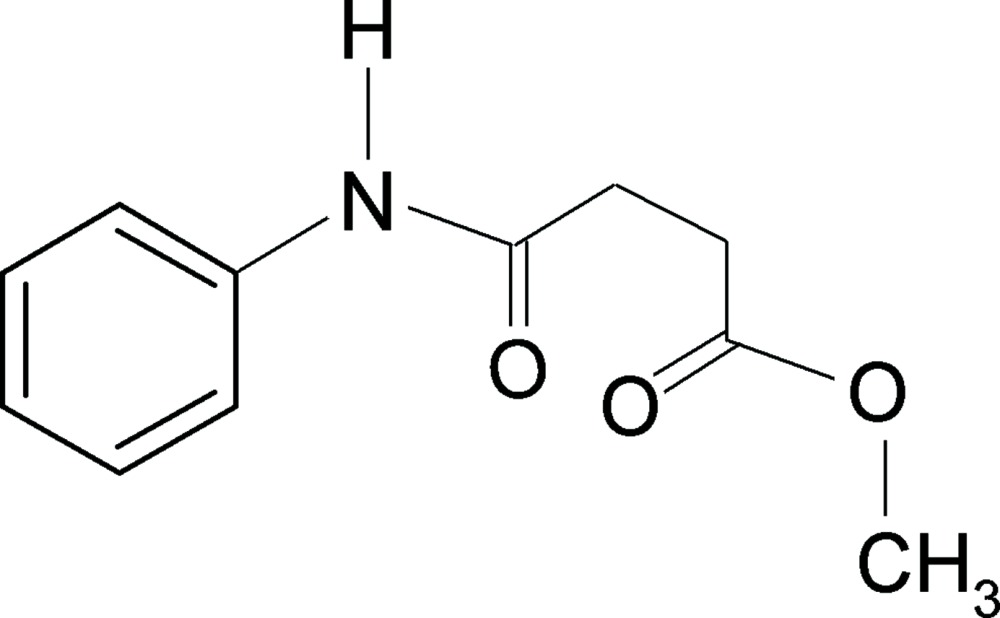


## Experimental

### 

#### Crystal data

C_11_H_13_NO_3_
*M* *_r_* = 207.22
Orthorhombic, 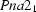

*a* = 15.973 (2) Å
*b* = 12.600 (1) Å
*c* = 5.2438 (9) Å
*V* = 1055.4 (2) Å^3^
*Z* = 4
Mo *K*α radiation
μ = 0.10 mm^−1^
*T* = 299 K
0.50 × 0.12 × 0.08 mm

#### Data collection

Oxford Diffraction Xcalibur diffractometer with a Sapphire CCD detector
Absorption correction: multi-scan (*CrysAlis RED*; Oxford Diffraction, 2009 ▶) *T*_min_ = 0.954, *T*_max_ = 0.992
2584 measured reflections
1184 independent reflections
774 reflections with *I* > 2σ(*I*)
*R*_int_ = 0.023

#### Refinement

*R*[*F* ^2^ > 2σ(*F* ^2^)] = 0.049
*wR*(*F* ^2^) = 0.096
*S* = 1.13
1184 reflections
140 parameters
1 restraint
H atoms treated by a mixture of independent and constrained refinement
Δρ_max_ = 0.11 e Å^−3^
Δρ_min_ = −0.11 e Å^−3^

### 

Data collection: *CrysAlis CCD* (Oxford Diffraction, 2009 ▶); cell refinement: *CrysAlis RED* (Oxford Diffraction, 2009 ▶); data reduction: *CrysAlis RED*; program(s) used to solve structure: *SHELXS97* (Sheldrick, 2008 ▶); program(s) used to refine structure: *SHELXL97* (Sheldrick, 2008 ▶); molecular graphics: *PLATON* (Spek, 2009 ▶); software used to prepare material for publication: *SHELXL97*.

## Supplementary Material

Crystal structure: contains datablocks I, global. DOI: 10.1107/S160053680904690X/pk2203sup1.cif

Structure factors: contains datablocks I. DOI: 10.1107/S160053680904690X/pk2203Isup2.hkl

Additional supplementary materials:  crystallographic information; 3D view; checkCIF report

## Figures and Tables

**Table 1:** Hydrogen-bond geometry (Å, °)

*D*—H⋯*A*	*D*—H	H⋯*A*	*D*⋯*A*	*D*—H⋯*A*
N1—H1*N*⋯O2^i^	0.85 (4)	2.20 (4)	3.036 (4)	170 (3)

Symmetry code: (i) 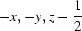.

**Fig. 1.**
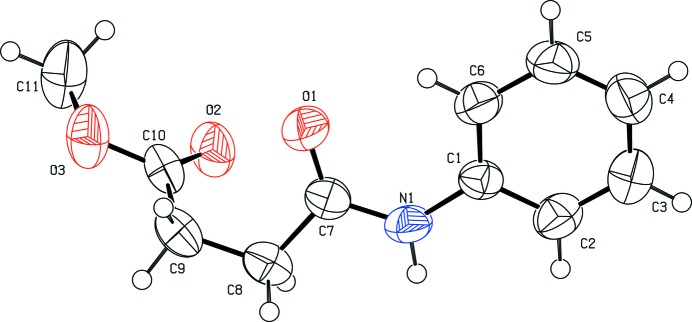
Molecular structure of the title compound, showing the atom labelling scheme. The displacement ellipsoids are drawn at the 50% probability level. The H atoms are represented as small spheres of arbitrary radius.

**Fig. 2.**
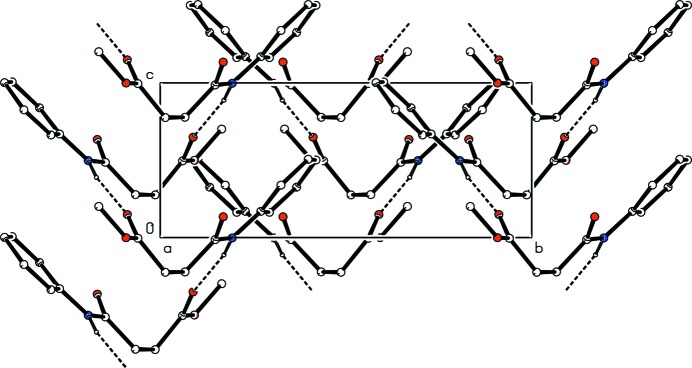
Molecular packing of the title compound with hydrogen bonding shown as dashed lines.

## supplementary crystallographic information

### Comment

Amides are of interest as conjugation between the nitrogen lone pair electrons and the carbonyl π-bond results in distinct physical and chemical properties. The amide moiety is also an important constituent of many biologically significant compounds. Thus the structural studies of amides are of interest see Gowda *et al.*, 2007, and references therein, 2009*a,b*; Jones *et al.*, 1990; as representative examples. As a part of studying the effect of ring and side chain substitutions on the solid state geometry of this class of compounds, we report herein the crystal structure of *N*-(phenyl)methylsuccinamate. The conformations of the N—H and C=O bonds in the amide fragment are *trans* to each other (Fig. 1). The side chain in the title compound is bent at C8 with C7—C8—C9—C10 torsional angle of 70.3 (4)°. The linking of molecules into a helical chain by N—H···O interactions (Table 1) is shown in Fig.2.

### Experimental

A solution of succinic anhydride (0.025 mole) in toluene (25 ml) was treated dropwise with a solution of aniline (0.025 mole) in toluene (20 ml) with constant stirring. The resulting mixture was stirred for about 1 h and set aside for an additional hour at room temperature for completion of the reaction. The mixture was then treated with dilute hydrochloric acid to remove the unreacted aniline. The resultant solid *N*-(phenyl)succinamic acid was filtered under suction and washed thoroughly with water to remove the unreacted succinic anhydride and succinic acid and was recrystallized from methanol. The recrystallized sample in methanol (20 ml) was treated with concentrated sulfuric acid (2 ml). The mixture was refluxed for 2 h and kept for slow evaporation at room temperature to obtain crystals of *N*-(phenyl)methylsuccinamate. The crystals were washed with water to remove sulfuric acid and dried. The purity of the compound was checked by elemental analysis and characterized by recording its infrared spectra. The single crystals used in X-ray diffraction studies were grown from methanolic solution by slow evaporation at room temperature.

### Refinement

The H atom of the NH group was located in a difference map and its position refined with N—H = 0.85 (4) Å. The other H atoms were positioned with idealized geometry using a riding model with C—H = 0.93–0.97 Å. Isotropic displacement parameters for the H atoms of the methyl group were set to 1.5 *U*_eq_ (parent atom), for the other H atoms equal to 1.2 *U*_eq_ (parent atom).

In the absence of significant anomalous dispersion effects, Friedel pairs were merged and the Δf" terms set to zero.

### Crystal data

**Table:**

C_11_H_13_NO_3_	*F*(000) = 440
*M**_r_* = 207.22	*D*_x_ = 1.304 Mg m^−^^3^
Orthorhombic, *P**n**a*2_1_	Mo *K*α radiation, λ = 0.71073 Å
Hall symbol: P 2c -2n	Cell parameters from 930 reflections
*a* = 15.973 (2) Å	θ = 2.6–27.5°
*b* = 12.600 (1) Å	µ = 0.10 mm^−^^1^
*c* = 5.2438 (9) Å	*T* = 299 K
*V* = 1055.4 (2) Å^3^	Rod, colourless
*Z* = 4	0.50 × 0.12 × 0.08 mm

### Data collection

**Table:**

Oxford Diffraction Xcalibur diffractometer with a Sapphire CCD detector	1184 independent reflections
Radiation source: fine-focus sealed tube	774 reflections with *I* > 2σ(*I*)
graphite	*R*_int_ = 0.023
Rotation method data acquisition using ω and φ scans	θ_max_ = 26.4°, θ_min_ = 2.6°
Absorption correction: multi-scan (*CrysAlis RED*; Oxford Diffraction, 2009)	*h* = −19→16
*T*_min_ = 0.954, *T*_max_ = 0.992	*k* = −10→15
2584 measured reflections	*l* = −6→5

### Refinement

**Table:**

Refinement on *F*^2^	Primary atom site location: structure-invariant direct methods
Least-squares matrix: full	Secondary atom site location: difference Fourier map
*R*[*F*^2^ > 2σ(*F*^2^)] = 0.049	Hydrogen site location: inferred from neighbouring sites
*wR*(*F*^2^) = 0.096	H atoms treated by a mixture of independent and constrained refinement
*S* = 1.13	*w* = 1/[σ^2^(*F*_o_^2^) + (0.0356*P*)^2^ + 0.1073*P*] where *P* = (*F*_o_^2^ + 2*F*_c_^2^)/3
1184 reflections	(Δ/σ)_max_ = 0.032
140 parameters	Δρ_max_ = 0.11 e Å^−^^3^
1 restraint	Δρ_min_ = −0.11 e Å^−^^3^

### Special details

**Table:**

Experimental. CrysAlis RED (Oxford Diffraction, 2009) Empirical absorption correction using spherical harmonics, implemented in SCALE3 ABSPACK scaling algorithm.
Geometry. All e.s.d.'s (except the e.s.d. in the dihedral angle between two l.s. planes) are estimated using the full covariance matrix. The cell e.s.d.'s are taken into account individually in the estimation of e.s.d.'s in distances, angles and torsion angles; correlations between e.s.d.'s in cell parameters are only used when they are defined by crystal symmetry. An approximate (isotropic) treatment of cell e.s.d.'s is used for estimating e.s.d.'s involving l.s. planes.
Refinement. Refinement of *F*^2^ against ALL reflections. The weighted *R*-factor *wR* and goodness of fit *S* are based on *F*^2^, conventional *R*-factors *R* are based on *F*, with *F* set to zero for negative *F*^2^. The threshold expression of *F*^2^ > 2σ(*F*^2^) is used only for calculating *R*-factors(gt) *etc*. and is not relevant to the choice of reflections for refinement. *R*-factors based on *F*^2^ are statistically about twice as large as those based on *F*, and *R*- factors based on ALL data will be even larger.

### Fractional atomic coordinates and isotropic or equivalent isotropic displacement parameters (Å^2^)

**Table:**

	*x*	*y*	*z*	*U*_iso_*/*U*_eq_	
O1	0.12956 (15)	0.16946 (18)	0.1342 (6)	0.0713 (7)	
O2	0.13057 (14)	−0.08851 (18)	0.1486 (6)	0.0664 (7)	
O3	0.26104 (16)	−0.09216 (19)	−0.0009 (6)	0.0751 (8)	
N1	−0.00368 (18)	0.1938 (2)	−0.0025 (7)	0.0556 (8)	
H1N	−0.039 (2)	0.171 (3)	−0.112 (7)	0.067*	
C1	−0.03455 (19)	0.2709 (2)	0.1695 (7)	0.0465 (8)	
C2	−0.1192 (2)	0.2940 (3)	0.1633 (9)	0.0629 (10)	
H2	−0.1534	0.2593	0.0466	0.076*	
C3	−0.1535 (2)	0.3670 (3)	0.3259 (10)	0.0702 (11)	
H3	−0.2105	0.3815	0.3178	0.084*	
C4	−0.1043 (3)	0.4192 (3)	0.5015 (10)	0.0676 (10)	
H4	−0.1278	0.4675	0.6148	0.081*	
C5	−0.0201 (3)	0.3984 (3)	0.5061 (10)	0.0692 (10)	
H5	0.0139	0.4339	0.6222	0.083*	
C6	0.0152 (2)	0.3251 (3)	0.3392 (8)	0.0619 (10)	
H6	0.0726	0.3127	0.3426	0.074*	
C7	0.0728 (2)	0.1478 (2)	−0.0132 (8)	0.0499 (8)	
C8	0.0820 (2)	0.0666 (3)	−0.2223 (7)	0.0602 (10)	
H8A	0.0730	0.1009	−0.3856	0.072*	
H8B	0.0391	0.0128	−0.2016	0.072*	
C9	0.1666 (2)	0.0136 (3)	−0.2240 (7)	0.0629 (10)	
H9A	0.1726	−0.0260	−0.3817	0.076*	
H9B	0.2096	0.0681	−0.2224	0.076*	
C10	0.1815 (2)	−0.0602 (2)	−0.0043 (8)	0.0528 (9)	
C11	0.2848 (2)	−0.1655 (3)	0.2016 (10)	0.0906 (16)	
H11A	0.2554	−0.2313	0.1799	0.136*	
H11B	0.2708	−0.1353	0.3640	0.136*	
H11C	0.3440	−0.1783	0.1941	0.136*	

### Atomic displacement parameters (Å^2^)

**Table:**

	*U*^11^	*U*^22^	*U*^33^	*U*^12^	*U*^13^	*U*^23^
O1	0.0574 (14)	0.0822 (16)	0.0745 (17)	0.0086 (12)	−0.0170 (17)	−0.0237 (19)
O2	0.0594 (14)	0.0779 (16)	0.0619 (15)	0.0023 (13)	0.0225 (17)	0.0105 (18)
O3	0.0616 (17)	0.0773 (16)	0.0863 (18)	0.0099 (13)	0.0306 (18)	0.0165 (19)
N1	0.0538 (19)	0.0599 (17)	0.0530 (18)	−0.0006 (14)	−0.0167 (17)	−0.0114 (19)
C1	0.055 (2)	0.0429 (17)	0.0418 (18)	0.0002 (16)	−0.004 (2)	0.0039 (19)
C2	0.053 (2)	0.067 (2)	0.069 (2)	−0.0030 (18)	−0.010 (3)	−0.002 (3)
C3	0.057 (3)	0.072 (3)	0.081 (3)	0.0072 (19)	0.005 (3)	0.004 (3)
C4	0.076 (3)	0.065 (2)	0.062 (2)	0.011 (2)	0.008 (3)	−0.003 (3)
C5	0.081 (3)	0.065 (2)	0.061 (2)	0.005 (2)	−0.016 (3)	−0.015 (2)
C6	0.059 (2)	0.063 (2)	0.064 (2)	0.0056 (18)	−0.016 (2)	−0.008 (2)
C7	0.056 (2)	0.0482 (18)	0.0455 (19)	−0.0040 (17)	0.000 (2)	0.001 (2)
C8	0.072 (2)	0.064 (2)	0.045 (2)	−0.0006 (19)	0.001 (2)	−0.003 (2)
C9	0.075 (2)	0.067 (2)	0.047 (2)	−0.003 (2)	0.018 (2)	−0.004 (2)
C10	0.057 (2)	0.0481 (18)	0.053 (2)	−0.0006 (17)	0.018 (2)	−0.008 (2)
C11	0.073 (3)	0.093 (3)	0.106 (4)	0.022 (2)	0.024 (3)	0.022 (3)

### Geometric parameters (Å, °)

**Table:**

O1—C7	1.222 (4)	C4—H4	0.9300
O2—C10	1.196 (4)	C5—C6	1.393 (5)
O3—C10	1.333 (4)	C5—H5	0.9300
O3—C11	1.458 (5)	C6—H6	0.9300
N1—C7	1.354 (4)	C7—C8	1.507 (5)
N1—C1	1.415 (4)	C8—C9	1.508 (5)
N1—H1N	0.85 (4)	C8—H8A	0.9700
C1—C6	1.375 (4)	C8—H8B	0.9700
C1—C2	1.383 (4)	C9—C10	1.500 (5)
C2—C3	1.369 (5)	C9—H9A	0.9700
C2—H2	0.9300	C9—H9B	0.9700
C3—C4	1.377 (6)	C11—H11A	0.9600
C3—H3	0.9300	C11—H11B	0.9600
C4—C5	1.370 (5)	C11—H11C	0.9600
			
C10—O3—C11	116.7 (3)	O1—C7—C8	122.7 (3)
C7—N1—C1	129.4 (3)	N1—C7—C8	114.1 (3)
C7—N1—H1N	115 (2)	C7—C8—C9	113.1 (3)
C1—N1—H1N	115 (2)	C7—C8—H8A	109.0
C6—C1—C2	118.4 (3)	C9—C8—H8A	109.0
C6—C1—N1	123.5 (3)	C7—C8—H8B	109.0
C2—C1—N1	118.0 (3)	C9—C8—H8B	109.0
C3—C2—C1	121.2 (4)	H8A—C8—H8B	107.8
C3—C2—H2	119.4	C10—C9—C8	114.3 (3)
C1—C2—H2	119.4	C10—C9—H9A	108.7
C2—C3—C4	120.6 (4)	C8—C9—H9A	108.7
C2—C3—H3	119.7	C10—C9—H9B	108.7
C4—C3—H3	119.7	C8—C9—H9B	108.7
C5—C4—C3	118.7 (4)	H9A—C9—H9B	107.6
C5—C4—H4	120.6	O2—C10—O3	123.3 (4)
C3—C4—H4	120.6	O2—C10—C9	126.3 (3)
C4—C5—C6	120.9 (4)	O3—C10—C9	110.4 (3)
C4—C5—H5	119.5	O3—C11—H11A	109.5
C6—C5—H5	119.5	O3—C11—H11B	109.5
C1—C6—C5	120.1 (3)	H11A—C11—H11B	109.5
C1—C6—H6	120.0	O3—C11—H11C	109.5
C5—C6—H6	120.0	H11A—C11—H11C	109.5
O1—C7—N1	123.2 (4)	H11B—C11—H11C	109.5
			
C7—N1—C1—C6	10.3 (6)	C1—N1—C7—O1	−0.8 (6)
C7—N1—C1—C2	−170.7 (4)	C1—N1—C7—C8	178.8 (3)
C6—C1—C2—C3	−1.6 (6)	O1—C7—C8—C9	1.7 (5)
N1—C1—C2—C3	179.4 (4)	N1—C7—C8—C9	−177.9 (3)
C1—C2—C3—C4	−0.3 (6)	C7—C8—C9—C10	70.2 (4)
C2—C3—C4—C5	1.6 (6)	C11—O3—C10—O2	0.2 (5)
C3—C4—C5—C6	−0.9 (6)	C11—O3—C10—C9	−179.4 (3)
C2—C1—C6—C5	2.2 (5)	C8—C9—C10—O2	8.9 (5)
N1—C1—C6—C5	−178.8 (4)	C8—C9—C10—O3	−171.5 (3)
C4—C5—C6—C1	−1.0 (6)		

### Hydrogen-bond geometry (Å, °)

**Table:**

*D*—H···*A*	*D*—H	H···*A*	*D*···*A*	*D*—H···*A*
N1—H1N···O2^i^	0.85 (4)	2.20 (4)	3.036 (4)	170 (3)

Symmetry codes: (i) −*x*, −*y*, *z*−1/2.
